# A comprehensive review and comparison of existing computational methods for protein function prediction

**DOI:** 10.1093/bib/bbae289

**Published:** 2024-06-20

**Authors:** Baohui Lin, Xiaoling Luo, Yumeng Liu, Xiaopeng Jin

**Affiliations:** College of Big Data and Internet, Shenzhen Technology University, Shenzhen, Guangdong 518118, China; Guangdong Provincial Key Laboratory of Novel Security Intelligence Technologies, Shenzhen, Guangdong, China; College of Computer Science and Software Engineering, Shenzhen University, Shenzhen, Guangdong 518061, China; College of Big Data and Internet, Shenzhen Technology University, Shenzhen, Guangdong 518118, China; College of Big Data and Internet, Shenzhen Technology University, Shenzhen, Guangdong 518118, China

**Keywords:** protein function prediction, sequence-based methods, 3D structure-based methods, PPI network-based methods and hybrid information-based methods

## Abstract

Protein function prediction is critical for understanding the cellular physiological and biochemical processes, and it opens up new possibilities for advancements in fields such as disease research and drug discovery. During the past decades, with the exponential growth of protein sequence data, many computational methods for predicting protein function have been proposed. Therefore, a systematic review and comparison of these methods are necessary. In this study, we divide these methods into four different categories, including sequence-based methods, 3D structure-based methods, PPI network-based methods and hybrid information-based methods. Furthermore, their advantages and disadvantages are discussed, and then their performance is comprehensively evaluated and compared. Finally, we discuss the challenges and opportunities present in this field.

## Introduction

**Figure 1:**
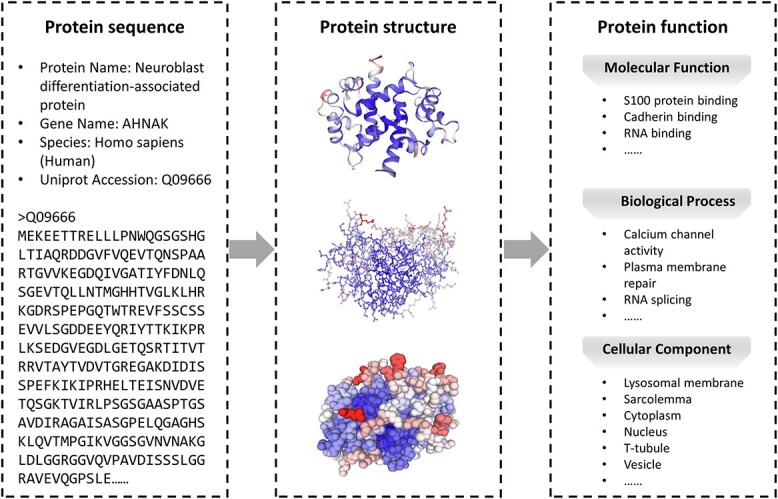
A schematic view of the ‘sequence-structure-function’ paradigm. Sequence refers to the arrangement of amino acids in a protein. Structure refers to the three-dimensional arrangement of a protein’s atoms. Function refers to the particular role a protein plays within an organism [8–10]. Generally, a protein sequence determines its structure, which, in turn, determines its function.

Proteins are vital constituents of living systems, playing diverse biological roles such as catalyzing chemical reactions, participating in signal transduction and maintaining cellular structure. Proteins must undergo multiple cellular steps to fulfill their functions and engage in cellular activities [1, 2]. The amino acid residues in a protein sequence undergo spatial transformation, leading to the formation of a three-dimensional structure. This is followed by the establishment of protein–protein interaction (PPI) networks through non-covalent forces [3], regulating both structure and function. Subsequently, PPI networks assemble into protein complexes [4, 5], which serve as molecular machines for realizing protein functions and executing life activities. It is noteworthy that not all proteins require complex formation and some can independently execute their functions. In protein research, the paradigm of ‘sequence-structure-function’ is followed. Currently, protein function is standardized by the Gene Ontology (GO) [6, 7], which encompasses three aspects: biological process ontology (BPO), molecular function ontology (MFO) and cellular component ontology (CCO), as shown in Figure 1.

Exploring protein function prediction holds profound significance in comprehending both intracellular and extracellular biological processes and uncovering the correlation between protein diversity and evolution. Additionally, protein function prediction plays a crucial role in elucidating gene functions, comprehending gene regulatory networks and guiding the functional annotation of uncharacterized proteins [11, 12]. Accurate prediction of protein function supports drug discovery and development by facilitating the creation of drugs targeting specific proteins, thereby enhancing treatment efficacy [12]. In personalized medicine [13], understanding the individual variations in protein function is essential for evaluating disease risk and devising individualized treatment strategies. In summary, protein function prediction serves as a crucial tool driving the progress of life science and medicine, thus contributing significantly to human health and sustainable development.

Traditional biological experiments exhibit high accuracy in determining protein function. However, conducting these experiments involves costly equipment and materials, intricate implementation steps, as well as substantial time and labor expenses [14]. Moreover, biological experiments require operations within living cells, and achieving accurate results requires multiple repetitions and analyses. Due to the limitations of traditional biological experiments, computational methods employing machine learning and deep learning techniques are widely utilized and exhibit robust performance [15–17]. In contrast to traditional experiments, computational methods demonstrate superior scalability and the ability to concurrently manage large-scale protein sample data, thereby enhancing research efficiency. Furthermore, computational methods can reduce research costs and decrease reliance on costly experimental equipment, as well as minimize time and labor requirements.

The Critical Assessment of Function Annotations (CAFA) Challenge [18] is a challenging competition in the field of bioinformatics designed to assess and advance the development of methods for protein function prediction. The challenge is organized by the international bioinformatics research community and attracts researchers from all over the world. Briefly, CAFA organizers provide a large number of protein sequences and participants are required to predict the function of these proteins using their computational methods, and these predictions are compared with the actual annotations to assess the accuracy of the computational methods. The CAFA challenge provides a platform for researchers to evaluate and compare their computational methods for predicting protein function. In addition, CAFA challenge has become an important competition in the field of bioinformatics, where researchers can learn about the advantages and disadvantages of various current function prediction algorithms and promote the development of protein function prediction methods [18–20].

Several review papers [21–23] have been proposed, summarizing existing computational methods. However, an updated and more comprehensive review is highly required for the following reasons: (i) Outdated classification: Sleator *et al*. [21] only classified protein function prediction methods as sequence-based and structure-based, while ignoring other bioinformatic data, such as PPI networks and Interpro. (ii) Lack of comprehensiveness: Shehu *et al*. [22] focuses on different categories based on the type of data used but lacks a discussion of the techniques employed for each category of method. (iii) Lack of comparison: xisting reviews categorize protein function prediction methods and describe the analyses, but they fall short in providing a comparative analysis of performance results computed on the same benchmark dataset.

In this review, we provide a comprehensive and up-to-date overview of existing computational methods for protein function prediction, focusing primarily on methods proposed in recent years. Initially, we introduce the databases used for predicting protein function. Subsequently, we propose a new categorization method to classify computational methods into four categories. Following that, we conduct a fair evaluation of the performance of each category of computational methods using the same benchmark dataset. Finally, we discuss future directions and challenges, exploring opportunities in this research area.

## Databases

In recent decades, owing to the development of this important filed, several databases have been constructed, including Uniprot [24], GO [25], GOA [26], STRING [27], InterPro [28], Research Collaboratory for Structural Bioinformatics Protein Data Bank (RCSB PDB) [29] and AlphaFold [30]. A summary of these protein databases is shown in Table 1.

**Table 1:** The summary of protein databases.

**Database**	**Version**	**Description$^{a}$**	**Website**
Uniprot [24]	January 2024	Swiss-Prot(570,830) TrEMBL(249,751,891)	https://www.uniprot.org/
GO [25]	17 January 2024	Biological Process(2,833,885) Molecular Function(2,459,729)	
		Cellular Component(2,362,323)	https://geneontology.org/
GOA [26]	v219	Electronic GO Annotations(1,372,042,474) Manual GO Annotations(5,916,798)	https://www.ebi.ac.uk/GOA/
STRING [27]	v12.0	Proteins(59,309,604) Organisms(12,535)	https://string-db.org/
InterPro [28]	v98.0	Homologous Superfamily(3,446) Family(21,942) Domain(14,053) Repeat(374) Site(953)	http://www.ebi.ac.uk/interpro/
RCSB PDB [29]	19 March 2024	PDB Structures(217,387) Computed Structure Models(1,068,577)	https://www.rcsb.org/
AlphaFold [30]	v2.0	Organisms(48) Predicted Structures(564,446)	https://alphafold.ebi.ac.uk/

$^{a}$ The protein database contains various types of data along with their respective quantities.

UniProt [24] is a widely utilized protein database, comprising various significant sections, including UniProtKB, UniRef and UniParc. UniProt release 2024_01 comprises over 250 million sequence records. The manually curated and reviewed entries section of UniProt is known as UniProtKB/Swiss-Prot, containing approximately 570 000 sequences. The unreviewed section is known as UniProtKB/TrEMBL and contains about 249 million sequences. Additionally, UniRef [31, 32] provides protein sequence clusters at three identity levels: 100, 90 and 50%. UniParc [33] provides a set of all known protein sequences, which stores all new and updated protein sequences from a variety of sources for comprehensive coverage. To avoid redundancy, each unique protein sequence is stored only once using a stable protein identifier [33, 34].

The GO [25] is a comprehensive resource for gene function information, where functional features used to describe gene function are classified into three categories: biological process (BP), molecular function (MF) and cellular component (CC). GO release 2024_01_17 comprises over 7 million annotations, 1 million gene products and 5000 species. Additionally, GO is available in three different versions on the download page(https://geneontology.org/docs/download-ontology/), including ‘go-basic’, ‘go’ and ‘go-plus’.

The Gene Ontology Annotation (GOA) [26] provides high-quality GO annotations for proteins in the UniProt Knowledgebase (UniProtKB). GOA employs two distinct GO annotation methods: electronic and manual [35]. Currently, the total count of GOA annotations exceeds 1.377 billion, with approximately 1.372 billion electronic GO annotations and only about 5.9 million manual GO annotations. Specifically, over 99 percent of GO annotations in the GOA database are made through electronic annotation methods.

STRING [27] is a database of known and predicted PPIs that integrates interaction data from five major sources, including genomic context predictions, high-throughput lab experiments, conserved-expression, automated textmining and previous knowledge in databases. STRING provides interaction scores between proteins, reflecting the strength of their connections in biological networks. The STRING database release 12.0 covers about 6 million proteins from over 10 000 organisms.

InterPro [28] is a comprehensive database that integrates 13 member databases: CATH-Gene3D [36], the Conserved Domains Database [37], HAMAP [38], PANTHER [39], Pfam [40], PIRSF [41], PRINTS [42], PROSITE Patterns [43], PROSITE Profiles [43], SMART [44], the Structure–Function Linkage Database [45], SUPERFAMILY [46] and TIGRFAMs [47]. Moreover, each member database contains focused characterization data. For example, the Pfam database primarily gathers, categorizes and annotates information related to protein domains.

The RCSB PDB [29] is a specialized database for storing information about the 3D structures of biological macromolecules. Currently, the RCSB.org portal provides over 200 000 experimentally determined PDB structures, with more than 120 000 eukaryotic protein structures and over half originating from humans. Additionally, approximately 1 million computed structure models (CSM) from AlphaFold DataBase [48] and ModelArchive are available.

AlphaFold [30] is a high-precision protein structure prediction database co-developed by DeepMind and EMBL-EBI, and in release v2.0, it contains more than 200 million entries that including the proteomes of 48 key organisms, providing broad coverage of UniProt. For protein structures not included in the database, structure prediction can be performed using the source code (https://github.com/google-deepmind/alphafold/).

In addition to the above databases that are currently widely used for protein function prediction studies, there are a number of other very important thermodynamic databases. For example, PINT [49], ProNIT [50], ProCaff [51] and PLD [52], these thermodynamic databases provide a wealth of thermodynamic information on protein interactions, which is an important reference value for understanding protein functions and interaction mechanisms.

## Methods

Over the past decades, numerous computational methods have been proposed to predict protein function. These methods can be categorized into four groups based on the type of information they utilize: sequence-based methods, 3D structure-based methods, PPI network-based methods and hybrid information-based methods. It’s important to note that these categories are not strictly different, and there is overlap and correlation between them. For example, some computational methods based on structure or PPI information also use sequence information to predict protein function.

### Sequence-based methods

**Figure 2:**
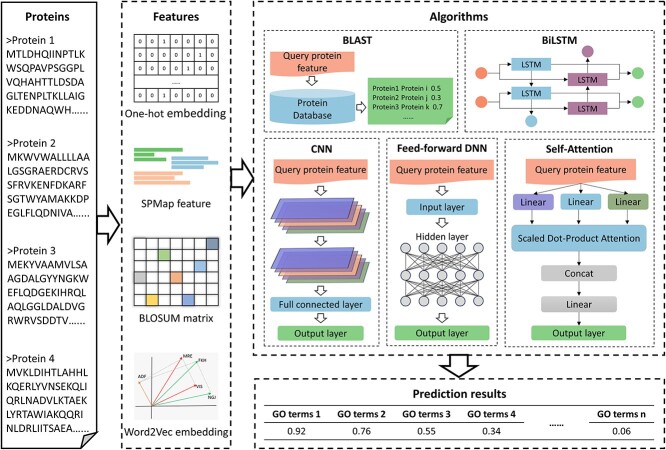
The flowchart of sequence-based methods. First, features are extracted from the sequence by different methods. Second, the extracted features are fed into some algorithm. Finally, the function of unknown proteins is predicted by the trained model, and the probability of their belonging to a specific GO term is calculated. However, BLAST tool searches the database for sequences similar to the target protein, eliminating the need to extract features from the sequence.

Sequence-based methods focus on the protein sequence to predict protein function by extracting potential features related to function from it. These methods delve deeper into the information contained in the amino acid sequences and attempt to capture subtle features related to protein function that are embedded within them. The flowchart of sequence-based methods is shown in Figure 2.

BLAST [53] is a widely employed tool for searching databases to find sequences similar to the target protein, thereby inferring potential functions of the target protein. However, relying solely on sequence alignment tools to predict protein function lacks high accuracy. Therefore, many methods have begun incorporating neural networks. In order to select the best protein feature representation, DEEPred [54] conducted separate deep neural network model training experiments using three different feature types, including subsequence profile map (SPMap) [55], pseudo amino acid composition (PAAC) [56] and conjoint triad [57]. The results show that the prediction performance with SPMap feature was the best. Consequently, DEEPred utilizes the SPMap features which extracted from the sequences and employs them as input for the multi-task feed-forward deep neural networks model to predict protein function. Combined with the prediction results from convolutional neural networks, DeepGOPlus [58] improves prediction accuracy by searching similar proteins with known functions. Compared to the previous model DeepGO [59], DeepGOPlus removes PPI features yet improves the prediction performance. In addition, it overcomes the sequence length limitation and replaces the amino acid trigram embedding layer with one-hot encoding. Drawing inspiration from the idea that proteins can be represented as a language, HiFun [60] embeds protein sequences using the BLOSUM62 matrix [61] and the FastText embedding. Vectors based on the BLOSUM62 matrix are fed into the convolutional layer to extract latent features, while matrices based on the FastText embedding are fed into an architecture consisting of convolutional neural network (CNN) and bidirectional long and short-term memory with self-attentive architecture (BiLSTM) [62]. The feature vectors extracted from the CNN are passed to the BiLSTM two hidden layers, which utilise the preceding and succeeding contextual information to enhance the feature representation [63]. In addition, HiFun performs the self-attention mechanism on the output of the BiLSTM layer to estimate the importance of amino acids.

These methods utilizes protein sequences solely for protein function prediction and plays a important role in discovering functions of new proteins. Several research experiments [64] have demonstrated that methods relying on protein sequences can significantly improve the prediction of MFO in functional categories. However, many proteins are similar in function but not in sequence, potentially leading to poorer predictive performance for proteins with low sequence similarity.

### 3D structure-based methods

**Figure 3:**
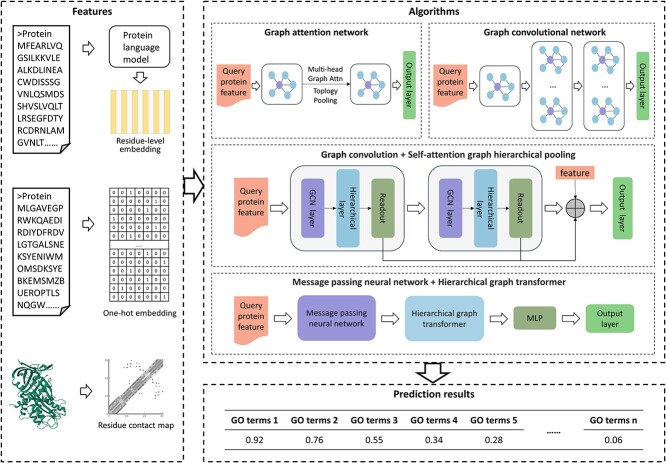
The flowchart of 3D structure-based methods. First, the feature extraction methods can be categorized into three types. (i) Extracting sequence embedding from sequences. (ii) Transforming structures into contact maps [65] and obtaining residue-level features. Second, the extracted features are fed into some algorithm. Finally, the function of unknown proteins is predicted by the trained model, and the probability of their belonging to a specific GO term is calculated.

3D structure-based methods focus on protein structure features. Protein structures are often translated into contact maps from which features closely related to function are captured. It’s important to note that protein sequence is frequently used as one of the input features in these methods. For proteins whose function is predominantly influenced by structure features, 3D structure-based methods have more pronounced advantages. The flowchart of 3D structure-based methods is shown in Figure 3.

DeepFRI [66] utilizes graph convolutional networks (GCN) [67] as its core technology. Firstly, it uses sequences from the protein family database (Pfam) [40] to pre-train the language model with long and short-term memory (LSTM-LM) [68] to extract residue-level features of the sequences. Secondly, it transforms protein structures into contact maps. Both are used as inputs to the GCN. The features learned by LSTM-LM from sequences and learned by GCN from contact maps significantly improve the performances of protein function prediction. Additionally, this method can also predict protein structure. In contrast to DeepFRI, GAT-GO [69] uses graph attention networks (GAT) [70] instead of traditional GCN. Each GAT layer is followed by a topological pooling layer [71] to perform topology-aware downsampling and a global pooling layer at the end. In addition, GAT-GO uses inter-residue contacts predicted by RaptorX [72] instead of the native contact maps used by DeepFRI. GAT-GO integrates predicted residue contact maps and CNN-generated sequence representations as GAT inputs, and combines GAT-generated representations with protein-level sequence embeddings to predict functional annotations. HEAL [73] is also compared to DeepFRI. The authors first trained the model HEAL-PDB using proteins from the Protein Data Bank (PDB) [74], which showed comparable performance to DeepFRI. The performance of HEAL was then significantly improved by incorporating protein structures predicted by AlphaFold2 [30]. HEAL first constructs graphs for each protein based on sequence node features and contact maps, which are fed into an architecture consisting of a message passing neural network and a hierarchical graph transformer. Then, supervised and comparative learning [75] are used to optimise the network. Struct2GO [76] takes both sequence and structure information as inputs. It utilises the SeqVec [77] model to extract sequence features and the hierarchical graph pooling [78] model based on the self-attention mechanism to extract features from structures predicted by AlphaFold2 [30]. To maximize the utilization of structure information, Struct2GO employs the Node2vec [79] algorithm to generate amino acid level embeddings in the protein structure network. These embeddings are used as the initial node features for the graph pooling model. Compared with the experimentally determined protein structures, AlphaFold2 [30] provides abundant high-resolution structure information, significantly improving model prediction accuracy.

These methods integrate protein structure alongside the protein sequence to substantially improve the accuracy of protein function prediction. For proteins with high similarity, it is difficult to distinguish minor functional differences using only sequence features, and combining structure features can help overcome this problem. However, the drawback of such methods is that they focus on protein structure features and ignore interactions between proteins.

### P‌PI network-based methods

**Figure 4:**
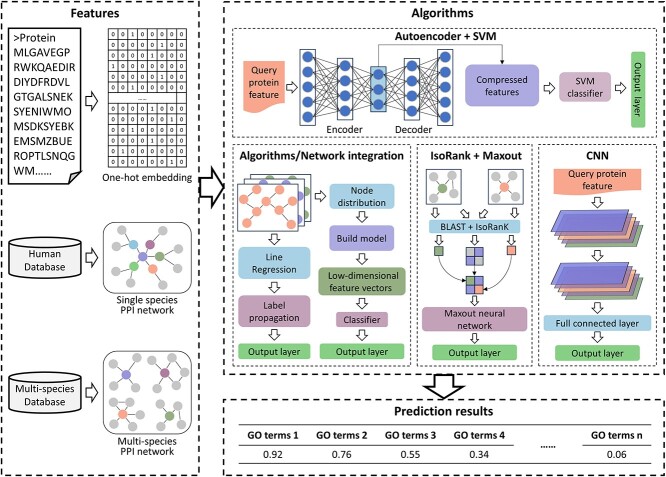
The flowchart of PPI network-based methods. First, the feature extraction methods can be categorized into three types. (i) Extracting sequence embedding from sequences. (ii) Obtaining single-species or multi-species PPI networks from the STRING database. Second, the extracted features are fed into some algorithm. Finally, the function of unknown proteins is predicted by the trained model, and the probability of their belonging to a specific GO term is calculated.

PPI network-based methods focus on PPI networks. Similar to 3D structure-based methods, protein sequence can also be utilized as one of the input features in these methods. The flowchart of PPI network-based methods is shown in Figure 4.

GeneMANIA [80] is a network integration algorithm that combines linear regression algorithm, which constructs composite functional association networks from multiple protein data sources, and label propagation algorithm, which predicts functional annotations for these networks. It utilizes a distinct optimization strategy compared to other functional association network methods [81, 82], emphasizing the adjustment of network weights and discriminant values. Moreover, GeneMANIA integrates an improved Gaussian field label propagation algorithm [83] to improve functional prediction accuracy. Similar to the GeneMANIA algorithm, Mashup [84] is a network integration framework that utilizes network diffusion to obtain the distribution of each node and captures correlation information with other nodes. Then, the topological information of each node is represented as low-dimensional spatial vectors, and finally these vector representations are used as inputs for functional prediction. Mashup improves prediction accuracy for two main reasons: one is to analyze the topology of each network individually, and the other is to convert topology information into a compact low-dimensional representation. With the wide application of deep learning techniques, a network fusion method named deepNF [85] has been proposed, which is constructed based on multimodal deep autoencoder (MDA). Autoencoder is a special type of neural network that consists of an encoding part and a decoding part. It has been shown to effectively remove noise [86]. deepNF converts heterogeneous networks into vector representations by constructing positive pointwise mutual information (PPMI) matrices that capture network information using random walk with restarts (RWR) method. Subsequently, it extracts compact low-dimensional features from the networks represented by the PPMI matrices via the MDA middle layer and trains SVM classifiers with the features. NetQuilt [87] uses the IsoRank [88] algorithm to calculate similarity scores between proteins of the same and different species separately, and then constructs the IsoRank similarity matrices. Subsequently, it combines the IsoRank matrices of all species into a new matrix that is used as input to the neural network with a maxout activation function [89]. The above methods integrate multiple types of single-species PPI networks into a single kernel or compact low-dimensional representation, while NetQuilt integrates the homology information of multiple-species and the global alignment of PPI networks into a meta-network profile. Based on the IsoRank algorithm, NetQuilt significantly outperforms methods based on single-species PPI networks by integrating similarity scores from multiple-species networks. DeepGO [59], a previous version of DeepGOPlus, uses multi-layer neural networks to learn features for protein function prediction from protein sequences and PPI networks. Because only a small number of proteins have such network information, DeepGOPlus removes the PPI network information and instead improves prediction performance by integrating a sequence similarity-based method.

These methods primarily relies on PPI networks. It has been demonstrated that proteins with interactions in PPI networks often exhibit similar functions [90], making it feasible to predict protein functions using PPI networks. However, predicting protein function solely based on PPI networks has limitations, as the data generated by high-throughput techniques may contain noise [91], potentially affecting result accuracy.

### Hybrid information-based methods

**Figure 5:**
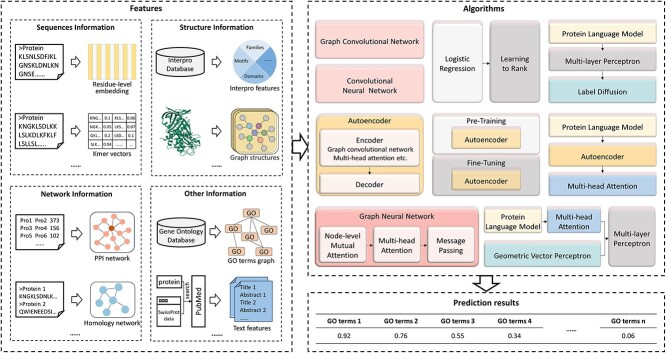
The flowchart of hybrid information-based methods. First, features can be extracted from four different types of information. (i) In sequence, extracting sequence embedding or residue-level vectors. (ii) In 3D structure, transforming structures into contact maps and obtaining residue-level features. (iii) In PPI networks, obtaining PPI networks from the STRING database. (iv)In InterPro features, obtaining features from the InterPro database. (v) Others, constructing GO term graphs, constructing homology networks based on sequences or query the literature for proteins and use them as textual features. Second, the extracted features are fed into some algorithm. Finally, the function of unknown proteins is predicted by the trained model, and the probability of their belonging to a specific GO term is calculated.

Hybrid information-based methods emphasize the combination of various information types, such as integration of protein sequences, protein structures, PPI networks or InterPro features and other integration methods. The CAFA challenge [18] has shown that the effective integration of diverse information types can genuinely improve the accuracy of protein function prediction [18–20]. Currently, hybrid information stands out as a major trend in the development of this field. The flowchart of hybrid information-based methods is shown in Figure 5.

#### Integration of sequences, structures, PPI or InterPro

GOLabeler [92] uses the learning to rank (LTR) [93] framework to integrate five types of information, including GO term frequencies, sequence similarity, amino acid triad frequencies, ProFET features [94] and InterPro features [95]. The candidate GO terms are then ranked, and a ranked list of GO terms as the final output. The advantage of GOLabeler is the use of the LTR framework, making it possible to integrate any classifier into the framework as a new component. NetGO [96] maintains GOLabeler’s LTR [93] framework and introduces a new component, Net-KNN, which further improves prediction performance by using STRING’s extensive network information. The basic idea of Net-KNN is similar to one of the components in GOLabeler, BLAST-KNN, with the difference being that Net-KNN uses the association scores in the network instead of sequence similarity as in BLAST-KNN. Besides LTR framework, neural network models are also commonly used. SDN2GO [97] employs an integrated deep learning model for protein function prediction. It utilizes three distinct neural network modules to extract features from protein sequences, protein domains and PPI networks, respectively. Subsequently, these features are integrated into a weighted classifier, comprising a three-layer non-fully-connected network that learns features from the three different sources to optimize the weights. Additionally, SDN2GO builds a sub-model SN2GO, in which features from protein domains are excluded. The experiment results show that incorporating protein domain information can significantly improve prediction performance, especially in aspects of BPO. Graph2GO [64] is a multimodal graph-based model that builds two graphs for PPI networks and sequence similarity networks, respectively. Attributes such as protein sequences, subcellular locations and protein domains serve as nodes in the two graphs. Subsequently, potential features are learned from the nodes in the graphs using the variational graph auto-encoder [98]. This architecture learns vector representations from both the network structure and node attributes, which can be applied not only to predict protein function but also to other tasks involving biological networks, such as predicting interactions between proteins and predicting protein complexes. DeepGraphGO [99] is a multi-species graph neural network-based method. It first utilizes the InterProScan [100] tool to generate InterPro feature vectors for proteins and combines PPI network to use the InterPro feature vectors as node features in the network. Higher-order information in the network is then captured by multiple graph convolutional layers. Additionally, the advantage of DeepGraphGO is that it employs a multi-species strategy that utilizing protein data from all species to train a single model, which not only saves time and computational resources, but also improves the generalization of the model. CFAGO [101] cross-fuse PPI networks and protein attributes in two stages based on multi-head attention mechanism [102]. The first step is pre-training, where an autoencoder is employed to learn hidden features within the PPI network and protein attributes. As mentioned previously, the autoencoder have the ability to ignore noise in the data. The second step is fine-tuning, wherein the pre-trained encoder is combined with a two-layer fully connected neural network to predict protein function. Additionally, ablation experiments indicate that pre-training contributes more to performance enhancement than the attention mechanism does. Recently, it has been shown that the protein structure information predicted by AlphaFold2 [30] significantly improves the accuracy of function prediction. PredGO [103] consists of three important modules: using the pre-trained protein language model ESM-1b [104] to extract protein sequence features, employing a graph neural network with geometric vector perceptron [105] to extract features from the protein structure predicted by AlphaFold2 [30], and utilizing a protein fusion layer based on the multi-head attention mechanism to fuse sequence features and PPI features, generating fusion features. Then, the fusion features and structure features are concatenated for functional prediction. Additionally, the ablation experiments demonstrate that structure information improves performance in aspects of MFO and CCO, while PPI information improves performance in aspects of BPO. Combining sequence, structure and PPI information, enables a more comprehensive improvement in prediction performance.

#### Other integration methods

Besides the information utilized by the previously described methods, various additional information has been used to predict protein function. Compared to the previous version of NetGO [96], NetGO 2.0 [106] integrates protein literature information from SwissProt [107] (LR-Text) and sequence information extracted by recurrent neural network (Seq-RNN) into the LTR [93] framework. As a component of NetGO, LR-ProFET, had minimal impact on overall performance and was removed in NetGO 2.0, but the overall performance was improved. In contrast to the previously discussed methods, the following methods use GO term information. PANDA2 [108] employs graph neural networks to model the GO-directed acyclic graph [109] and integrates them with sequence features generated based on protein language model. Additionally, PANDA2 utilizes sequence alignment features based on PSI-BLAST, DIAMOND and Priority Score, as well as PAAC sequence attribute features. Moreover, the node attributes are iteratively updated in each network block of PANDA2 and knowledge can be learned from neighborhoods or receptive fields by stacking three network blocks. SPROF-GO [110] utilizes DIAMOND [111] to construct sequence homology networks instead of PPI networks. Initially, it extracts sequence embeddings from a pre-trained protein language model ProtT5 [112] and feeds them into two multilayer perceptrons (MLP) to learn attention scores and hidden embedding matrices. Subsequently, the hidden embeddings are weighted averaged based on the attention scores, which are then input into the output MLP to get the initial prediction. Additionally, SPROF-GO applies a hierarchical learning strategy. During the testing phase, a homology network is constructed based on the training set, and both the initial prediction and the homology network are input into the mark-diffusion algorithm to obtain the final prediction. PFresGO [113] encodes proteins as sequence embeddings using pre-trained protein language model ProtT5 [112]. Subsequently, it generates residue-level embeddings by combining one-hot sequence embeddings with sequence embeddings reduced in dimensionality by autoencoder. Additionally, PFresGO utilizes Anc2vec [114] to construct GO term embeddings based on the self-attention mechanism. These two types of embeddings are fused to calculate the probability of GO term based on the multi-head attention mechanism. The results indicate that even without using information beyond the sequence, the hierarchical structure of the GO graph can effectively improve prediction performance. Another method that utilize GO term information is HNetGO [115], which employs the DIAMOND [111] tool to calculate sequence similarity for building similarity networks. It integrates sequence features extracted based on pre-trained protein language model SeqVec [77], GO term relevance information and PPI networks to build heterogeneous networks. The networks are utilized to extract node features by using GNN model based on attention mechanism for predicting protein functions. Additionally, HNetGO innovatively constructs the heterogeneous information networks, which effectively integrate sequence and PPI network information, enabling the modelling of complex relationships between proteins and GO terms.

Through effectively fusing information from multiple sources, these methods can capture the diversity features of protein from multiple aspects, thereby improving the performance of functional prediction. Additionally, these methods can partially alleviate the noise effect inherent in information from a single data source. However, fusing information from different data sources usually necessitates more sophisticated algorithms or techniques to extract feature, particularly on extensive datasets, which may lead to increased consumption of computational resources and time. Furthermore, some proteins may lack source information of certain features, resulting in a lack of comprehensive fusion information for these proteins. Therefore, correctly handling missing data is a major challenge for such methods.

### Web servers and stand-alone tools

Because of the importance of protein function prediction, some web servers and stand-alone tools have been established, which are listed in Table 2. By using these servers or tools, we can easily reproduce these methods and make new predictions. From this table, we can clearly see the information on which these methods are based and the algorithms used. These have an impact on their performance results.

**Table 2:** The summary of web servers and stand-alone tools for protein function prediction.

Category$^{a}$	Method	Year	Website	Information	Algorithm
S	BLAST [53]	1990	https://blast.ncbi.nlm.nih.gov/Blast.cgi	Sequence	Sequence similarity search
	DEEPred [54]	2019	https://github.com/cansyl/DEEPred	Sequence	Multi-task feed-forward deep neural network
	DeepGOPlus [58]	2020	http://deepgoplus.bio2vec.net/	Sequence	Convolutional neural network and Sequence similarity search
	HiFun [60]	2023	http://www.unimd.org/HiFun	Sequence	Convolutional neural network and Bidirectional long short-term memory with self-attention mechanism
D	DeepFRI [66]	2021	https://beta.deepfri.flatironinstitute.org	Sequence and Structure	Protein language model and Graph convolutional network
	GATGO [69]	2022	∖	Sequence and Structure	Protein language model and Graph attention network
	HEAL [73]	2023	https://github.com/ZhonghuiGu/HEAL	Sequence and Structure	Protein language model, Message passing neural network and Hierarchical graph transformer
	Struct2GO [76]	2023	https://github.com/lyjps/Struct2GO	Sequence and Structure	Protein language model, Graph convolution and Attention-based graph pooling mechanism
P	GeneMANIA [80]	2008	∖	PPI	Linear regression and Label propagation algorithms
	Mashup [84]	2016	https://mashup.csail.mit.edu/	PPI	Network integration
	deepNF [85]	2018	https://github.com/VGligorijevic/deepNF	PPI	Autoencoder and Support vector machine
	DeepGO [59]	2018	http://deepgo.bio2vec.net/	Sequence and PPI	Convolutional neural network
	NetQuilt [87]	2021	https://github.com/nowittynamesleft/NetQuilt	PPI	IsoRank algorithm and Maxout neural network
H1	GOLabeler [92]	2018	∖	Sequence and InterPro	Logistic regression and Learning to rank
	NetGO [96]	2019	∖	Sequence, PPI and InterPro	Logistic regression and Learning to rank
	SDN2GO [97]	2020	https://github.com/Charrick/SDN2GO	Sequence, PPI and InterPro	Convolutional neural network
	Graph2GO [64]	2020	https://integrativeomics.shinyapps.io/graph2go/	Sequence, PPI and InterPro	Autoencoder and Graph convolutional network
	DeepGraphGO [99]	2021	https://github.com/yourh/DeepGraphGO	PPI and InterPro	Graph convolutional network
	CFAGO [101]	2023	http://bliulab.net/CFAGO/	PPI and InterPro	Autoencoder, Pre-training and Multi-head attention mechanism
	PredGO [103]	2023	http://predgo.denglab.org/	Sequence, PPI and Structure	Protein language model, Geometric vector perceptron and Multi-head attention mechanism
H2	NetGO 2.0 [106]	2021	∖	Sequence, PPI, InterPro and Literature	Logistic regression and Learning to rank
	PANDA2 [108]	2022	http://dna.cs.miami.edu/PANDA2/	Sequence and GO term	Protein language model and Graph convolutional network
	SPROF-GO [110]	2023	http://bio-web1.nscc-gz.cn/app/sprof-go	Sequence and Homology network	Protein language model, Self-attention mechanisms and Label diffusion algorithm
	PFresGO [113]	2023	https://github.com/BioColLab/PFresGO	Sequence and GO term	Protein language model, Autoencoder and Multi-head attention mechanism
	HNetGO [115]	2023	https://github.com/BIOGOHITSZ/HNetGO	PPI, Homology network and GO term	Protein language model, Graph neural network and Multi-head attention mechanism

$^{a}$ ‘S’ represents sequence-based methods, ‘D’ represents 3D structure-based methods, ‘P’ represents PPI network-based methods and ‘H’ represents hybrid information-based methods. ‘H1’ represents integration of sequences, structures, PPI or InterPro. ‘H2’ represents other integration methods.

We have systematized existing papers that explicitly state prediction methods. Currently, computational methods for predicting protein function can be divided into two main categories: integrated prediction methods and individual prediction methods. Integrated prediction methods train one model to predict MFO, BPO and CCO labels in three categories, such as Naïve [19], BLAST [53], DeepGOPlus [58], Mashup [84], deepNF [85] and PANDA2 [108]. Individual prediction methods train three separate models each for predicting one category of labels. It is worth noting that GeneMANIA [80] employs the Gaussian label propagation algorithm for label prediction, which is different from the above two categories of methods.

## Discussion and conclusion

As introduced and discussed above, many computational methods for protein function prediction have been proposed due to their importance. In this section, we provide a comprehensive comparison of these methods on a widely used benchmark dataset.

### Datasets

**Figure 6:**
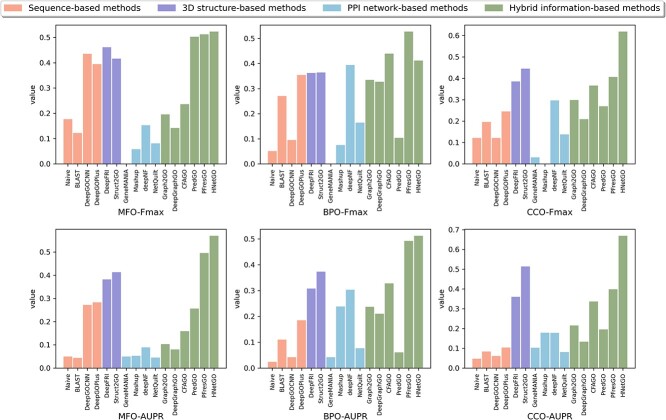
Performance comparison of four categories computational methods.

The datasets we used for performance comparison strictly followed the standards of the CAFA challenge [18]. To ensure the accuracy and reliability of the experimental results, we used manually curated and reviewed proteins in UniProtKB/Swiss-Prot [116] and annotations are propagated using the hierarchical structure of GO [6], as the annotations of unreviewed proteins are electronically made (evidence code: IEA), which may contain potential errors. In addition, according to CAFA, annotations with evidence codes (EXP, IDA, IPI, IMP, IGI, IEP, TAS and IC) are considered experimental, and therefore we selected proteins with experimental evidence codes. In the aspects of MFO, BPO and CCO, the number of training sets is 2747, 3197 and 5263, the number of validation sets is 503, 304 and 577, the number of testing sets is 719, 182 and 119, and the number of labels is 38, 45 and 35, respectively [24–30, 101]. The performance results of the various computational methods presented in Table 3 and Figure 6 are obtained from experiments conducted on this dataset.

**Table 3:** Performance comparison of different computational methods.

	**F$_{\max }$**			**AUPR**		
**Method** $^{a}$	**MFO**	**BPO**	**CCO**	**MFO**	**BPO**	**CCO**
Naive [19]	0.177	0.051	0.121	0.050	0.024	0.047
BLAST [53]	0.122	0.270	0.196	0.044	0.110	0.084
DeepGOCNN [58]	0.435	0.095	0.121	0.272	0.042	0.061
DeepGOPlus [58]	0.394	0.354	0.245	0.283	0.185	0.104
DeepFRI$^{b}$ [66]	0.461	0.362	0.385	0.382	0.308	0.360
Struct2GO [76]	0.416	0.364	0.445	*0.413*	*0.373*	0.514
GeneMANIA [80]	0.000	0.000	0.031	0.050	0.042	0.103
Mashup [84]	0.058	0.075	0.000	0.053	0.238	0.179
deepNF [85]	0.153	0.394	0.297	0.089	0.303	0.178
NetQuilt [87]	0.081	0.164	0.138	0.045	0.077	0.081
Graph2GO [64]	0.196	0.335	0.298	0.103	0.237	0.215
DeepGraphGO [99]	0.142	0.327	0.209	0.080	0.210	0.133
CFAGO [101]	0.236	0.439	0.366	0.159	0.328	0.337
PredGO [103]	*0.503*	0.104	0.269	0.256	0.061	0.195
PFresGO [113]	0.512	**0.527**	*0.406*	0.496	0.492	*0.399*
HNetGO [115]	**0.523**	*0.412*	**0.618**	**0.570**	**0.512**	**0.669**

The best performance is highlighted with bold font, and the performance ranked the second is highlighted with underline, and the performance ranked the third is highlighted with italic. $^{a}$In the implementation of these methods, the predictions for a protein are not counted under the following conditions. (i) Lack of structure data. (ii) Sequence lengths too long for input to the language model. $^{b}$The dataset built by DeepFRI does not contain sequences longer than 1000, so we filter out sequences from our dataset that are longer than 1000 for this method implementation.

### Evaluation

To evaluate the prediction accuracy of different methods from different perspectives, we select two different metrics, including F-max score (Fmax) and area under the precision-recall curve (AUPR). Fmax [117] is a protein-centered measure used in the CAFA challenge [19] and many protein function prediction studies, which takes values from 0 to 1, where 1 indicates the best performance of the model and 0 indicates the worst performance [19]. It is defined as following: 


(1) \begin{align*}& F_{\max }=\max_{\tau}\left\{\frac{2 \times p r(\tau) \times r c(\tau)}{p r(\tau)+r c(\tau)}\right\}\end{align*}

where pr($\tau $) and rc($\tau $) are the precision and recall in terms of threshold $\tau $, respectively, which are defined as following: 


(2) \begin{align*} & \operatorname{pr}(\tau)=\frac{1}{m(\tau)} \sum_{i=1}^{m(\tau)} \frac{\sum_{f} I\left(f \in P_{i}(\tau) \bigwedge f \in T_{i}\right)}{\sum_{f} I\left(f \in P_{i}(t)\right)} \end{align*}

(3) \begin{align*} & \operatorname{rc}(\tau)=\frac{1}{n} \sum_{i=1}^{n} \frac{\sum_{f} I\left(f \in P_{i}(\tau) \bigwedge f \in T_{i}\right)}{\sum_{f} I\left(f \in T_{i}\right)} \end{align*}

where *$f$* is a GO class, $P_{i}(\tau )$ is a set of predicted annotations for a protein *$i$* and threshold $\tau $, *$T_{i}$* is a set of true annotations for the protein, *m*(*$\tau $*) is the number of proteins we predict for at least one class and *$n$* is the total number of proteins and *$I(\bullet )$* is an indicator function.

In addition, AUPR is a function-centered measure used in many protein function prediction studies, which is also a reasonable metric for evaluating high levels of category imbalance in the predicted outcomes [118]. Both AUPR and AUROC are very general criteria for classification evaluation. However, because AUROC may not be sensitive enough in the case of classification imbalance, as it takes into account the balance between true positive and false positive rates, which can be affected by a large number of negative class samples. In contrast, AUPR better reflects the performance of the classifier in predicting positive cases, i.e. it is more able to penalize false positives [66, 92, 119]. It also takes values from 0 to 1, where 1 indicates the best performance of the model and 0 the worst performance [19].

### Performance comparison of four categories methods

Figure 6 shows the results of the four categories of methods on three aspects of GO. Overall, hybrid information-based methods outperforms the other three categories of methods in several metrics. For MFO and CCO aspects, 3D structure-based methods show more significant improvement compared to sequence-based methods, and for BPO aspects, PPI network-based methods show more significant improvement.

A more detailed summary of the performance results for different methods is shown in Table 3. It is evident that hybrid information-based methods achieve the best or second best results in terms of Fmax and AUPR measures. Specifically, PFresGO achieves the best performance in aspects of BPO with Fmax values of 0.527. Additionally, HNetGO achieves the best performance in aspects of MFO and CCO with Fmax value of 0.523 and 0.618, respectively. Moreover, HNetGO achieves the best performance in aspects of MFO, BPO and CCO with AUPR values of 0.570, 0.512 and 0.669, respectively. It is worth noting that due to the low proportion of training proteins in the PPI networks, GeneMANIA is unable to effectively propagate label information from training proteins to test proteins. Similarly, and Mashup also fails to effectively learn compact low-dimensional representations from the network. This results in some aspects of GO having a value of 0 for Fmax.

Hybrid information-based methods outperform other three categories of methods in several metrics, indicating that the strategy of integrating multi-source data information is reasonable and contributes to improving prediction accuracy. The reason for this is that hybrid information-based methods can effectively utilise data from different sources, such as sequence, structure, PPI, InterPro, GO terms, etc. These data are often complementary, providing different but relevant information that complements each other and enriches the understanding of protein function. Specifically, sequence provides information about amino acid composition and arrangement, forming the basis for inferring protein function. Structure provides information about the 3D structure and conformation of proteins, revealing important features such as the folding modes and the location of functional sites. In addition, PPI provides a global view of protein interactions in the organism, revealing the complex network structure of protein function regulation and signalling. InterPro provides multiple types of protein characterisation and GO terms provide detailed descriptions of the functional characteristics of proteins, respectively, which help to predict protein functions more accurately.

In order to further investigate the impact of sequence, structure and PPI on prediction performance, we excluded hybrid information-based methods. As evident from the results of the three categories, DeepFRI achieves the best performance in aspects of MFO with Fmax value of 0.461, and deepNF performs best in aspects of BPO with Fmax value of 0.394. In comparison, Struct2GO achieves the best performance in aspects of CCO with Fmax value of 0.445. Additionally, Struct2GO achieves the best performance in aspects of MFO, BPO and CCO with AUPR values of 0.413, 0.373 and 0.514, respectively. deepNF achieves comparable performance in aspects of BPO with AUPR values of 0.303. These results show that sequence and structure information contribute to improving performance in aspects of MFO and CCO, while PPI information contribute to improving performance in aspects of BPO. Specifically, MFO mainly describes the molecular function of a protein, while CCO describes the composition and structure of the cell in which the protein resides. Sequence information provides detailed information about the amino acid composition of a protein, and functionally relevant semantic information related to function can be extracted from the sequence. Structure information, on the other hand, provides more multifaceted information, including subcellular locations, protein domains and internal structural features. This information complements each other and reveals not only the molecular functions of proteins and their mechanisms of action inside and outside the cell, but also the localization and composition of proteins within the cell. In addition, BPO mainly describes the biological processes or activities in which proteins are involved, usually involving interactions and regulation among multiple proteins. PPI information provides information on protein interaction networks, signaling and metabolic regulation, revealing the overall role and function of proteins in biological processes. Therefore, sequence information, structure information and PPI information play different roles in protein function prediction tasks, which also reflects their different focuses and characteristics in the three aspects of MFO, BPO and CCO.

### Accuracy comparison of structure prediction methods

Since the precise structure often determines the function of a protein, we are naturally concerned about the accuracy of the structures used in 3D structure-based approaches. In this regard, deep learning methods have shown great potential. Among them, deep learning methods including AlphaFold [120] and RaptorX [121] have already achieved good results. In addition, we have chosen two other advanced deep learning methods ProSPr [122] and trRosetta [123] for comparison. RaptorX and trRosetta use deep residual neural networks (ResNet) as their core deep learning architecture. In contrast, the core component of AlphaFold and ProSPr is a deep CNN. Notably, the contact accuracy of these four methods on the CASP13 [124] test set was evaluated in a recent study [125], and the performance results of these four methods are summarized in detail in Table 4. It is obvious that AlphaFold shows the most excellent performance in all aspects. Because of this, most 3D structure-based methods choose to use AlphaFold-predicted protein structures as input features for protein function prediction, and the accuracy and reliability demonstrated by AlphaFold provides strong support for these methods. Using AlphaFold-predicted high-precision structures as input features, these methods can predict protein function more accurately.

**Table 4:** The contact accuracy of these four methods on the CASP13 test set.

**Method$^{a}$**	**Short(%)**	**Mid(%)**	**Long(%)**	**Avg(%)**
AlphaFold [120]	**95.13**	**87.12**	**72.53**	**84.93**
RaptorX [121]	92.47	84.65	69.46	82.19
ProSPr [122]	93.12	83.10	67.21	81.14
trRosetta [123]	92.73	84.77	69.30	82.27

The best performance is highlighted with bold font, and the performance ranked the second is highlighted with underline. $^{a}$ These experimental results in the table are referenced from associated literature [125].

### Accuracy of predicting unannotated proteins

**Figure 7:**
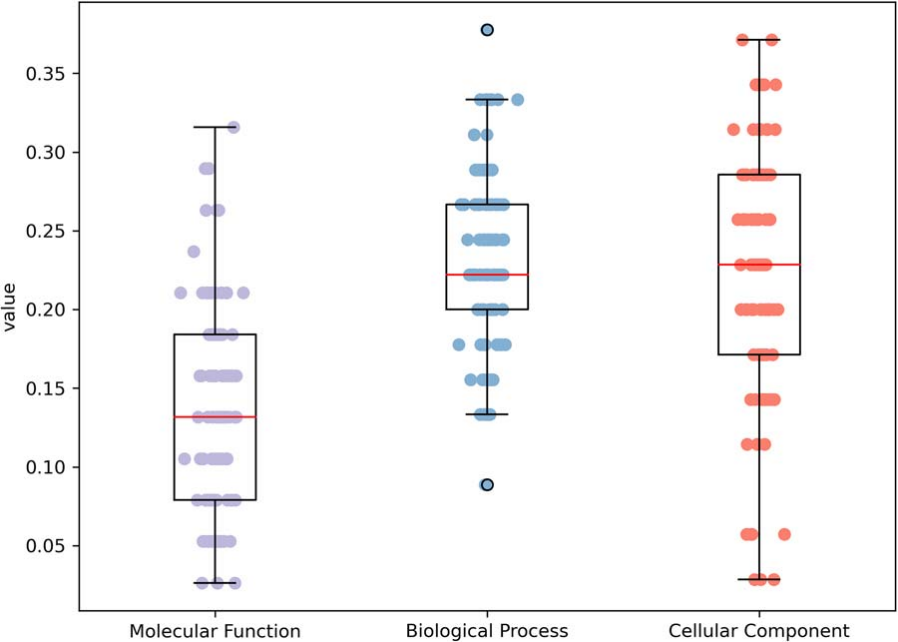
The accuracy of the best performing method on the unannotated protein set.

In order to assess the accuracy of the best-performing method in predicting unannotated protein functions, we selected the latest 100 human proteins from the Uniprot [24] database for prediction evaluation, and the results in Figure 7 show that the maximum accuracies in the three functional categories of MFO, BPO and CCO are 0.316, 0.378 and 0.371, respectively, and the average accuracies are 0.138, 0.229 and 0.220. Although the current best-performing computational methods do not achieve the desired accuracy, we need to note that these results are only predictive values and are subject to a degree of uncertainty. Furthermore, the assessment of accuracy is also affected by a degree of uncertainty, as the labels on which the assessment relies are based on computational methods for prediction and lack experimental validation. Therefore, we believe that computational methods using deep learning still have great potential for development in the field of unannotated protein function prediction.

### Problems and perspectives of protein function prediction

All the aforementioned methods have significantly contributed to the advancement of protein function prediction. However, there still exist some problems in this field. In this section, we will discuss these problems.

#### Benchmark datasets

Datasets are crucial for the construction and evaluation of predictive models. However, there are several problems with currently available protein datasets. (i) Very limited reviewed protein data. More than 99% of the proteins in the latest version of the Uniprot database still need to be reviewed [24]. The high cost of experimentally determining protein structures results in relatively limited coverage of structure data [126]. In addition, PPI data may contain noise and false positives [91, 127, 128], which could impact the accuracy and reliability of protein interactions. Therefore, more effort should be invested in collecting reliable protein data. (ii) Many protein function prediction methods have been proposed [15–17], but most trained and tested with different datasets. Models trained using data collected by one method may perform poorly on datasets built by other methods, making it difficult to directly and objectively compare the performance of different methods based on different test sets. In order to promote the development of the field, it is crucial to build an updated and more comprehensive benchmark dataset.

#### Feature extraction methods

Feature extraction methods are critical for protein function prediction. In the ‘Methods’ section, many feature extraction methods are presented, which are constructed based on different protein information. Specifically, (i) Based on sequence, where similarity information [129] is computed with the help of search tools or semantic information is extracted by the protein language models [77, 104, 112]. (ii) Based on structure, where the structure is transformed into contact maps, and then the residue-level features are obtained algorithmically. (iii) Based on PPI, where the PPI is represented as a graph structure, and then the features are extracted from the graph topology and network features. (iv) Based on InterPro, which integrates a variety of information. For example, binary features can be built using the presence or absence of subcellular locations [64] and protein domains [101]. From the results of the ‘Performance comparison of four categories methods’ section, we can see that the prediction performance can be significantly improved by combining the sequence, structure, PPI, InterPro and other forms of protein information. However, most protein function prediction methods rely on the effective integration of existing feature extraction methods to obtain feature information. Therefore, it is crucial to develop new feature extraction methods, which can provide more comprehensive and accurate representations of protein features.

#### Algorithms

The methods based on machine learning or deep learning achieve advanced performance in this field. The key to improving their performance is to find suitable algorithms. In recent years, the use of deep learning algorithms [130, 131] to predict protein function has become a hot topic of research in this field. For example, graph neural networks [132], autoencoders [133], attention mechanisms [102], etc., which show promise for improving the predictive performance. As more advanced deep learning algorithms are proposed and widely used in various fields. How to utilize or combine these deep learning models to build more accurate protein function predictors is one of the current important issues in this field. Currently, computational methods exist that are constructed based on data for a species with sufficient functional annotations. However, for species with sparse data and insufficient information, the utilization of established methods may result in poor prediction performance. To solve this problem, transfer learning [134] can be introduced into protein function prediction, which utilizes the existing species information to improve the predictive performance of new species information by different training strategies and mitigate the problem caused by sparse data and insufficient information.

**Key Points**

- Owing to the importance of protein function prediction in the field of bioinformatics, it is crucial to develop efficient and accurate computational methods to predict protein function.
- The databases of proteins are introduced and discussed, providing the useful information of these databases.
- The computational methods for protein function prediction are introduced, and their advantages and disadvantages are discussed. The existing web servers and stand-alone tools in the field are given.
- The performance of existing computational methods in this field is evaluated and compared based on a widely used benchmark dataset. Finally, some problems and perspectives in the field are discussed.

## Author Biographies

**Baohui Lin** is an undergraduate at the College of Big Data and Internet, Shenzhen Technology University, Shenzhen, Guangdong, China.

**Xiaoling Luo**, PhD, is an assistant professor at the College of Computer Science and Software Engineering, Shenzhen University, Shenzhen, China. She is also with Guangdong Provincial Key Laboratory of Novel Security Intelligence Technologies, Shenzhen, China. Her expertise is in deep learning, medical image processing and computer vision.

**Yumeng Liu**, PhD, is a lecturer at the College of Big Data and Internet, Shenzhen Technology University, Shenzhen, Guangdong, China. Her expertise is in bioinformatics, nature language processing and machine learning.

**Xiaopeng Jin**, PhD, is a lecturer at the College of Big Data and Internet, Shenzhen Technology University, Shenzhen, Guangdong, China. His expertise is in bioinformatics, nature language processing and machine learning.
